# Nanoparticle-Embedded Polymers and Their Applications: A Review

**DOI:** 10.3390/membranes13050537

**Published:** 2023-05-22

**Authors:** Nezar H. Khdary, Basha T. Almuarqab, Gaber El Enany

**Affiliations:** 1 King Abdulaziz City for Science and Technology, Riyadh 11442, Saudi Arabia; 2 Department of Physics, College of Science and Arts in Uglat Asugour, Qassim University, Buraydah 52571, Saudi Arabia; g.elenany@qu.edu.sa

**Keywords:** nanoparticules, membrane, composite membrane, nano-membrane, nanoparticle-embedded

## Abstract

There has been increasing interest in the study and development of nanoparticle-embedded polymeric materials and their applications to special membranes. Nanoparticle-embedded polymeric materials have been observed to have a desirable compatibility with commonly used membrane matrices, a wide range of functionalities, and tunable physicochemical properties. The development of nanoparticle-embedded polymeric materials has shown great potential to overcome the longstanding challenges faced by the membrane separation industry. One major challenge that has been a bottleneck to the progress and use of membranes is the balance between the selectivity and the permeability of the membranes. Recent developments in the fabrication of nanoparticle-embedded polymeric materials have focused on how to further tune the properties of the nanoparticles and membranes to improve the performance of the membranes even further. Techniques for improving the performance of nanoparticle-embedded membranes by exploiting their surface characteristics and internal pore and channel structures to a significant degree have been incorporated into the fabrication processes. Several fabrication techniques are discussed in this paper and used to produce both mixed-matrix membranes and homogenous nanoparticle-embedded polymeric materials. The discussed fabrication techniques include interfacial polymerization, self-assembly, surface coating, and phase inversion. With the current interest shown in the field of nanoparticle-embedded polymeric materials, it is expected that better-performing membranes will be developed soon.

## 1. Introduction

The development of nanoparticle-embedded polymeric membranes has had a huge impact on the material separation technologies that continue to support crucial industries such as the water industry, on the mitigation of the environmental crisis, on carbon dioxide capture, and on the scarcity of energy, among other significant areas affecting human needs and development. For these applications, polymeric membranes are used as selectively permeable membranes, allowing selected particles, ions, molecules, and other small particles to pass through, while blocking the passage of other particles [1]. Polymeric membranes are used today as separation media for different mixtures such as solid, liquid, and gaseous mixtures, as well as colloidal mixtures [2,3,4]. 

**Figure 1:**
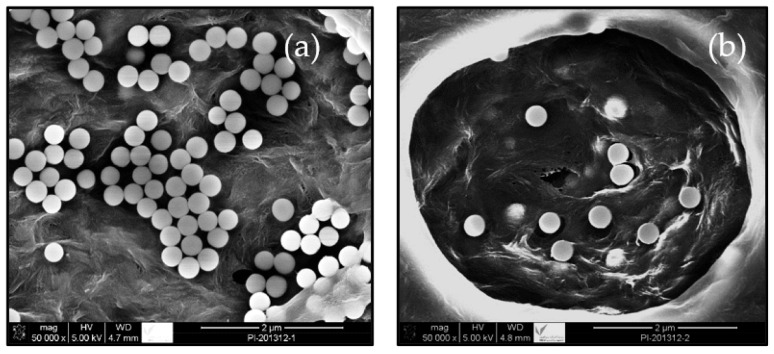
SEM images of PVDF polymer embedded with nanoparticles (**a**). A pore of the polymer membrane embedded with nanoparticles (**b**). In both images, the phase inversion technique was applied.

Polymeric membranes integrate technologies such as nanoparticle separation, ultrafiltration, microfiltration, pervaporation, gas separation, and reverse osmosis. The practical use of polymeric membrane technologies in the separation processes applied in various industries requires the continuous development of these materials to ensure their effectiveness, high permeability, stability, and optimal selectivity [5]. One of the most-used technologies to enhance polymeric membranes is the modification of a membrane using nanoparticles [6]. The use of this technology under certain conditions enhances a membrane’s resistance to fouling since, under regular use, membranes are susceptible to fouling, causing their performance to degrade over time. Significant improvements and modifications should be made to polymeric membranes before they can be used sustainably [7]. The incorporation of nanoparticles into polymeric membranes, when using different types of materials, has the potential to cause a synergistic effect [8]. The advanced industrial application of nanoparticle-embedded polymeric materials depends on the ability of researchers to develop materials with distinct and competitive specifications [9]. The performance of polymeric films has been improved via the addition of nanometals such as silver nanoparticles and gold nanoparticles, as well as other nanomaterials, particularly titanium dioxide, carbon nanotubes, and zinc oxide (i.e., TiO_2_, CNT, and ZnO). These materials are easily available, inexpensive, and environmentally friendly [10,11]. One of the major advantages of using nanoparticle-embedded polymer membranes is the possibility of regulating the mass transfer in such membranes by altering their surface properties to achieve the desired characteristics, hence avoiding the tradeoff between permeability and selectivity; see Figure 1 [12].

The scope of this review covers polymeric and nanoparticle-embedded polymeric materials’ fabrication and separation for various applications. 

## 2. Factors Affecting the Performance of Polymeric Membranes

Polymeric membranes comprise the most important membrane technology, and the performance of this membrane technology relies heavily on the membranes’ properties. The functioning of polymeric membranes is affected by various factors, such as material selection, membrane preparation, operation parameters, and membrane surface characteristics. 

Material selection: The choice of the membrane material is an important factor in determining the performance of the membrane. The properties of the membrane material, such as its chemical resistance, hydrophobicity, biocompatibility, and mechanical strength directly affect the membrane’s performance. Different membrane materials have different pore sizes and surface characteristics, factors which can be used to control the flux and selectivity of a membrane. Structural properties, such as crosslinking in the polymer membranes, determine membrane characteristics such as permeability and flow rate. These properties may be improved once nanoparticles are added to the polymer in specific proportions.

Membrane preparation: The type of membrane preparation process used is also an important factor in determining the membrane’s performance. Fabrication processes, such as extrusion, phase inversion, and casting, can be used to produce membranes with different pore sizes, surface characteristics, and hydrophobic/hydrophilic properties. 

Operation parameters: The operation parameters, such as the feed pressure, feed concentration, and crossflow velocity, can also affect the membrane’s performance. High feed pressures can clog the membrane, resulting in reduced permeability. High feed concentrations can also lead to fouling. 

Membrane surface characteristics: The surface characteristics of the membrane, such as the surface charge, hydrophobicity, and surface roughness, can also affect the membrane’s performance. Membranes with higher surface charges can cause electrostatic repulsions and reduce membrane fouling. In addition, hydrophobic membranes can reduce the water flux, while hydrophilic membranes can increase the water flux. Membranes with higher surface roughness can increase the membrane’s porosity and permeability. In addition, the incorporation of nanoparticles into the polymer can change the degree of hydrophilicity or hydrophobicity, depending on the nature of those particles and the functional groups attached to them. Attaching hydrophobic groups to the nanoparticles and doping polymers with them will increase their hydrophobic properties.

Each of these factors can have a significant impact on the membrane’s performance and they must be carefully considered when designing and operating the membrane system, especially when doped with nanoparticles. 

The performance of polymeric membranes is determined by the effectiveness of their separation performance, something which is mainly affected by the mass transfer process of the ions and materials in the mixtures. The process should be as efficient as possible, as described by Hagen–Poiseuille’s law, which describes the nature of the transport of particles through polymeric membranes [13]. The nature of operation of the polymeric membranes changes as the pores decrease. As the size of the membrane pores approaches the sub-nanometer scale, other mechanisms, such as the Donnan effect and confined mass transport effects, become increasingly significant in determining the behavior and performance of the polymeric membranes [14]. Membranes using reverse osmosis, pervaporation, and gas separation mechanisms will allow molecules to pass through them as guided by the mechanism of solution–diffusion. 

In the solution–diffusion mechanism, the rate of permeability through the polymeric membrane relies on the ability of the different particles to diffuse through the membrane pores [15]. Furthermore, other factors used conventionally to determine and evaluate the performance of polymeric membranes for the separation of particles include the rejection, permeability, flux, and selectivity of the membranes. Theoretically, membranes with high rejection and high flux, commonly measured as permeability, are considered to provide the lowest cost and energy consumption in material separation [16]. In practice, however, the most functional and economical polymeric membranes are amorphous and contain several chains of intertwined polymers. These factors would, in most cases, result in a reduction in pore size in the membrane, and simultaneously cause a reduction in the resistance to mass transport, resulting overall in the reduced effectiveness and accuracy of separation.

Due to this, there is usually a practical dilemma in the use of polymeric membranes, given the imbalance between the selectivity and permeability of a membrane, and technologists are required to shift in emphasis between the two. Most polymeric membranes tested have been shown to exhibit these characteristics, especially when separating organic molecules and inorganic gas molecules [17]. To combat the challenge above, the use of nanotechnology to modify membranes has been attempted to some degree of success, with researchers using nanomaterials that are generally inorganic in order to influence the permeability of the membrane walls. The nanomaterials used include graphene oxide (GO), carbon nanotubes (CNTs), metal–organic frameworks (MOFs), molecular sieves, nanoparticles made of metal and metal oxides, zeolites and transition metal dichalcogenide (TMD) nanosheets.

These nanomaterials have been useful in engineering separation membranes with enhanced properties [18,19,20,21]. The improvement of polymeric membranes by using nanomaterials occurs due to the formation of continuous, straight transport channels. Although the inorganic nanomaterials have shown evidence of greatly improving the separation performance of polymeric membranes, they have shown themselves to possess very poor compatibility with the polymeric matrices and are difficult to fabricate for large-scale production, a factor which inhibits their practical, industrial use. Consequently, technologists still face challenges in the fabrication of materials which improve the large-scale use of membranes via the utilization of nanomaterials with both higher selectivity and permeability [22].

Recent developments have indicated that there are polymeric nanomaterials with the desired chemical properties that could be used alternatively as they are not challenging to fabricate and a more compatible with the polymeric matrix [23]. These nanomaterials have been found to have chemical compositions that can be tuned to suit the required composition, possess higher compatibility with the polymeric matrix, and can easily be manufactured through the large-scale production of advanced separation media [24].

## 3. Development and Production of Nano-Based Polymeric Membranes

The development and production of nano-based polymeric membranes offer a promising approach to enhancing the performance of polymeric membranes in various applications. This technology has the potential to improve the efficiency, selectivity, and durability of membrane processes, leading to the development of more sustainable and cost-effective solutions. However, the performance of polymeric membranes is often limited by their relatively low selectivity and permeability. The development of nano-based polymeric membranes aims to overcome these limitations by incorporating nanomaterials into the polymer matrix to enhance the membrane properties. 

**Figure 2:**
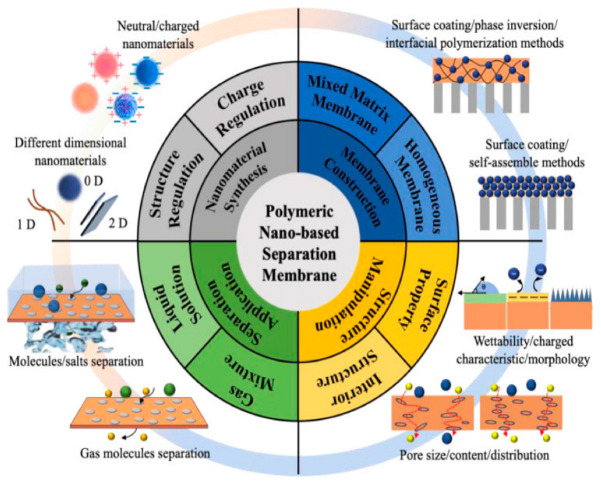
Application of polymeric nanomaterials and their nano-based membranes for structural modification and separation. Reprinted from reference [35].

As noted by Yang and Yang, the use of nanoparticle-embedded polymeric materials is in its early stages. Therefore, all the achievements and developments in this field ought to be compiled. In this section, the recent developments and the commonly used methods of fabricating nanoparticle-embedded polymeric materials will be discussed. The recent advancements in polymer science have contributed to the development of nanoparticle-embedded polymeric materials. These include protein nanoparticles, polyelectrolyte nanoparticles, hyperbranched polymers, and polymeric nanofibers among other separation materials [25]. We will discuss the fabrication of both nanoparticle-embedded polymeric materials and homogenous nano-polymeric membranes [26]. Table 1 and Figure 2 summarized some developed polymer membranes incorporating nanoparticles.

**Table 1:** Some examples of developing polymer membranes with nanoparticles.

Membrane	Materials	Filler Type	Modification Technique	The Purpose of Modification and Its Advantages	Applications	Ref.
PSF/diatomite composite.	Polysulfone (PSF).	Silicon Dioxide (SiO_2_).	Phase inversion.	Exhibit excellent hydrophilicity, large-pore voids, and low surface roughness.	Remove oil, dye, and pharmaceutical waste from water bodies by treating effluents.	[27]
Ultrafiltration membrane.	(PVDF)/(PAN).	SiO_2_ and TiO_2_	Phase inversion.	Improvement in flux and antifouling properties.	Extensively used in drinking water and wastewater.	[28]
PSF-PVP/LDH-Mt	PSF-PVP	Montmorillonite (Mt) modified with Mg-Fe double hydroxide layers (LDH-Mt).	Phase inversion.	The most hydrophilic materials, of the best thermal stability, and the highest surface roughness, induces a radical change in the architecture of pores. Modified membranes have an elongated, interconnecting pore structure.	Used for separation of an oil–water mixture.	[29]
Composite films of interconnected silica networks on the polyester fabric.	Polyester fabric.	Octadecyl-polysiloxane networked silica nanoparticles.	One-step dip-coating method	Exhibited higher surface roughness and the membrane surface increased the contact angle to 178 degrees and swiftly passed nonpolar organic solvents, with an outstanding oil separation efficiency of >99.5%.	Effective oil–water separation	[30]
PET/Pd nanoparticles.	Polyethylene terephthalate (PET).	Pd nanoparticles solution.	Carboxylation and nanoparticles deposition in porous polymeric membranes.	Increase in the selectivity of hydrogen in comparison to carbon dioxide (CO_2_) and nitrogen (N_2_) as carboxylation promotes H_2_ selectiveness.	Hydrogen separation.	[31]
PS/PP-FOTS-SiO_2_NPs	Polysulfone/polypropylene membrane.	Perfluorooctyltriethoxysilane modified silica nanoparticles (FOTS-SiO_2_NPs).	Phase separation process.	The oil flux and separation efficiencies obtained were 84.9 L/h·m^2^ and 83.4%, respectively. Efficient oil cleaning from polluted water sources.	Oil–water emulsion separations.	[32]
(PVDF)–ZnO composite membranes.	Polyvinylidene fluoride.	Zinc oxide (ZnO).	Blending process.	Confer substantial hydrophilic, photocatalytic, and antibacterial activities to polymeric membranes.	Wastewater treatments.	[33]
PVDF-HFP/SiO_2_.	PVDF-HFP polymer.	SiO_2_	Phase separation.	The porosity of the membrane was controlled, improving the mechanical properties of the membrane and excellent selectivity toward CO_2_.	CO_2_ Capturing and water purifications.	[34]

### 3.1. Nanoparticle-Embedded Polymeric Membranes Manufacturing Processes and Applications

Nanoparticle-embedded polymeric membranes are composite materials that combine the properties of polymers with the unique characteristics of nanoparticles, such as high surface area, high reactivity, and unique optical and magnetic properties. These materials have potential applications in various fields such as biomedical, environmental, and energy-related technologies. Figure 2 shows the application of polymeric nanomaterials and their nano-based membranes for the purposes of structural modification and separation. 

In the field of water treatment, these membranes can be used in desalination, water purification, wastewater treatment, and polluted air treatment. In gas separation, these membranes are used to separate gases such as hydrogen, helium, and methane. In addition to the above, they have uses in the medical and biomedical fields such as tissue engineering, drug delivery, and biosensors [36]. As a result, industries specializing in petrochemical, oil production, pharmaceutical products, and food processing are embracing emerging separation techniques to gain a competitive edge and protect consumers from substandard products [36].

One of the most common uses for hydrophobic hollow fiber membranes is in gas-liquid membrane contactors. The liquid and gas phases are separated by a hollow fiber membrane. This also serves as an interface and provides a vast contact surface. Hollow fiber membrane contactors have many advantages, including a high surface-area-to-volume ratio, linear scalability, design, and a wide range of possible applications [37]. However, the most significant difficulty faced by membrane contactors is the wetting of membranes due to the penetration of liquid adsorbent which results in a significant decrease in performance. Therefore, it is vital to improve the hydrophobicity and chemical stability of membranes in order to boost the efficiency of this equipment [38,39,40,41,42,43].

#### 3.1.1. Nano-Based Polymeric Mixed-Matrix Membranes (MMMs)

Polymeric nanomaterial-based mixed-matrix membranes (MMMs) possess good performance stability and demonstrate effective membrane separation due to their perfect compatibility and the dispersibility of the nanomaterials involved [44]. However, the permeability of the infused membranes is still lower than the permeability of the pure nanomaterials due to the higher resistance exhibited by the polymeric matrices [45]. Mixed-matrix membranes are created through the fusion of monomer solutions with the nanoparticles [3,46,47]. In this process, the two join reactively to form mixed-matrix membranes, commonly known as MMMs [48]. Occasionally, mixed-matrix membranes are commonly known as hybrid membranes since they comprise nanoparticles linked to polymers. MMMs constitute classical porous fillers, nonporous fillers, and carbon molecular sieves. Notably, MMMs are effective for gas separation due to the presence of polymers with indispensable processing capabilities. Mixed-matrix membranes have commonly been found to improve the separation of CO_2_ and CH_4_ gases. Despite developing innovative membranes, researchers have extensively researched in order to improve the gas permeability and polymer structure of these membranes in order to ease gas separation [49].

In addition, chemical engineers have developed several approaches to prepare MMMs for gas and gas separation in order to curb energy prices and reduce the emission of greenhouse gas. The pore size, pore distribution, and overall membrane properties can be tailored by controlling the manufacturing process. 

By choosing the appropriate process, membranes can be produced with a range of pore sizes and distributions to meet specific requirements. Further research is needed to optimize these processes and produce high-performance membranes for use in a variety of applications.

##### Sol-Gel Process

Sol-gel is the most essential of numerous methods that can be used to improve the hydrophobicity and chemical stability of polymeric membranes. A surface with high hydrophobicity can be fabricated by carefully controlling the various parameters that affect the synthesis of nanoparticles in the sol-gel process [50,51,52]. A hydrophobic polyetherimide hollow fiber membrane was created by Wang et al. by covering the surface of the membranes with fluorinated silica NPs using the sol-gel technique. The experimental findings from this study demonstrated that the hydrophobicity and chemical stability of these membranes were significantly enhanced after the coating of the materials with fluorinated silica nanoparticles [37,42]. The CH_3_-grafted silica NPs were incorporated into a polypropylene hollow fiber membrane to create a superhydrophobic composite hollow fiber membrane. This was then used to absorb CO_2_ with a membrane contactor. The results showed that protecting the polymeric membrane from liquid absorbents was another benefit of incorporating the CH_3_-g silica NPs into the surface of the polypropylene membrane [37].

Challenges such as nanoparticle agglomeration, incompatibility with the polymer matrix, and potential environmental and health risks associated with some types of nanoparticles must be addressed. Further research is needed to optimize the manufacturing processes and explore the full potential applications of nanoparticle-embedded polymeric membranes in different applications.

##### Electrospinning 

Electrospinning is a process in which a polymer solution is passed through a high-voltage field to produce nanofibers. Electrospinning is based on the principle of charge repulsion, whereby an electric field is applied to a polymer solution, causing the formation of charged droplets. These droplets are then spun into ultrafine fibers, which are collected on a grounded collector. The fibers are collected onto a collector and form a porous membrane. The electrospinning process is a versatile and cost-effective method for the production of polymeric nano-based membranes. The technology offers the potential to produce highly uniform porous membranes with tunable pore sizes and high surface areas, making it a promising solution for use in various applications, including water purification, gas separation, and biomedicine.

The process parameters, such as solution concentration, applied voltage, and spinning distance, have a significant impact on the morphology and properties of the resulting membrane. In recent years, various modifications have been made to the traditional electrospinning process to improve the quality of the membranes produced [53,54,55]. For example, the addition of surfactants and other additives to the polymer solution can improve the stability of the droplets and the uniformity of the fibers produced. Recent studies have reported the use of electrospinning to produce membranes with pore sizes which range from a few nanometers to several microns in size. These are ideal for use in a variety of applications, including molecular separation and filtration. Additionally, the technology has also been utilized to produce composite membranes by combining different polymers or incorporating inorganic materials into the fibers. Many of the recent studies in the field of electrospinning for the production of nanoparticle-embedded polymers cover areas such as the electrospinning of polyvinyl alcohol nanofiber membranes for water treatment applications [56] and the fabrication and characterization of electrospun polyvinylidene fluoride nanofiber membranes for water treatment [57].

##### Phase Inversion Process

**Figure 3:**
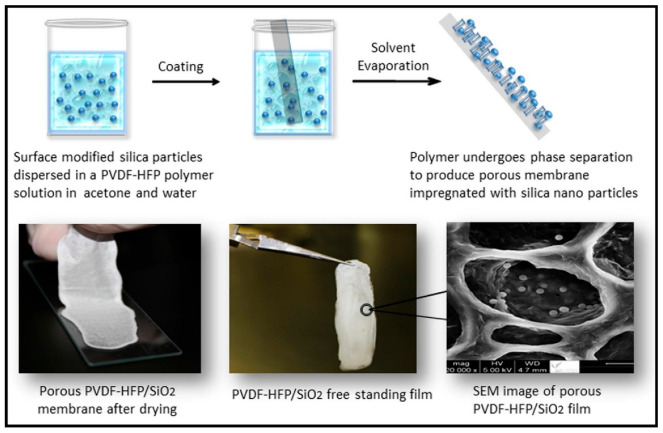
Phase Inversion process for making polymer nanocomposite, obtained with permission from Elsevier [34].

Phase inversion is one of the most widely used methods for producing nanoparticle-embedded polymers [58]. During this process, a polymer solution is cast onto a substrate and then subjected to a phase change that transforms the solution into a solid, porous membrane, as shown in Figure 3. This process can be controlled to produce membranes with precise pore size, porosity, and selectivity [59]. The factors of pore size and distribution can be controlled by adjusting the process parameters. The phase inversion process is an efficient and widely used method for producing nanoscale polymeric films. It has the potential to play an essential role in the development of new nanoparticle-incorporated membranes. One of the key advantages of the phase inversion process is its versatility and scalability, making it suitable for the production of membranes on a large scale. Additionally, the phase inversion process allows for the precise control of the membrane’s pore size, porosity, and selectivity, making it a highly desirable method for producing nanoparticle-embedded polymers [60]. In recent years, new phase inversion techniques have been developed to enhance the performance and durability of nanoparticle-embedded polymers produced through the phase inversion process. For example, non-solvent-induced phase inversion [61,62] uses a different approach to produce a gel-like structure. Conversely, supercritical fluid phase inversion [63] and thermally induced phase inversion [62] employ alternative methods to induce the phase change from a solution to a solid, porous membrane. These new techniques have shown promising results and have the potential to provide high-quality, high-performance nanoparticle-embedded polymers for use in a variety of applications. In addition, the parameters that influence the phase inversion process, including the choice of solvent and polymer, are under continuous investigation to optimize membrane production [63]. This includes casting conditions such as temperature and pressure. By manipulating these parameters, researchers aim to manufacture nanoparticle-embedded polymers with accurate pore size, porosity, and selectivity.

Phase inversion is the regulated transition of a polymer from its liquid phase to its solid phase. Precipitation from vapor phase, precipitation by controlled evaporation, thermally induced phase separation, and immersion precipitation are the four fundamental methods utilized to make phase inversion membranes. In the process, there is an exchange of non-solvent and solvent molecules, leading to the formation of a film that contains both a matrix and polymeric nano-fillers [64]. The film produced in this stage is then washed and subjected to post-treatment in order to further refine the matrix–filler material produced [65].

Polyethersulfone is commonly used to prepare mixed-matrix membranes due to its mechanical, thermal, and chemical capabilities. The phase inversion process is used to manufacture MMMs due to their ability to form a thin coating on a membrane. The membrane comprises a thin coating and a porous sublayer as these components improve gas molecules’ permeability and selectivity properties. Notably, the polymer membrane size is affected by temperature and pressure changes [66]. Hence, industrial experts introduce nanomaterials with a high permeation rate and morphological properties in order to improve the mechanical strength of membranes. Additionally, graphene nanosheets (GNs) have been shown to have a high water content for reasons of hybrid matrix membrane fabrication. Graphene nanosheets (GNs) accelerate the movement of mole molecules within the membrane. Occasionally, the process involves fabricating graphene nanosheets (GNs) with a mixture of four mixed-matrix membranes and pristine PES membranes through the phase inversion method [66]. In a previous study, the graphene nanosheets (GNs) used had weights of 0.01, 0.02, 0.03, and 0.04 wt%, and the homogeneous solution was subjected to a temperature of 50 °C for 6 h. The membranes were later immersed in deionized water at a temperature of 70 °C for 10 min. The solution was removed from the glass material and dipped in fresh deionized water at room temperature for less than 24 h. Finally, the membrane was dried and used in biogas production and oil manufacturing. [67].

Phase inversion was used to create ultrafiltration membranes using polyacrylonitrile (PAN) and polyvinylidene fluoride (PVDF) blends. These were then modified by the addition of silicon dioxide nanoparticles (SiO2) and titanium dioxide nanoparticles (TiO2) [47].

The chemistry of the dope polymer solution is drastically altered by the interaction between nanoparticles, polymer chains, and solvent molecules [47].

NPs, possessing large surface areas and an affinity for water, not only improve flow, and chemical and mechanical characteristics, but they also give antibacterial and antifouling qualities to membranes made from an extensively dope polymer solution using the phase inversion approach. The progressive rise in nano silica concentration may cause a shift in the rate of solvent-to-non-solvent exchange during phase inversion, resulting in a gradual change in membrane shape [47]. This type of membrane has been successfully used to purify conventional solvents, such as ethanol and isopropanol [68].

**Figure 4:**
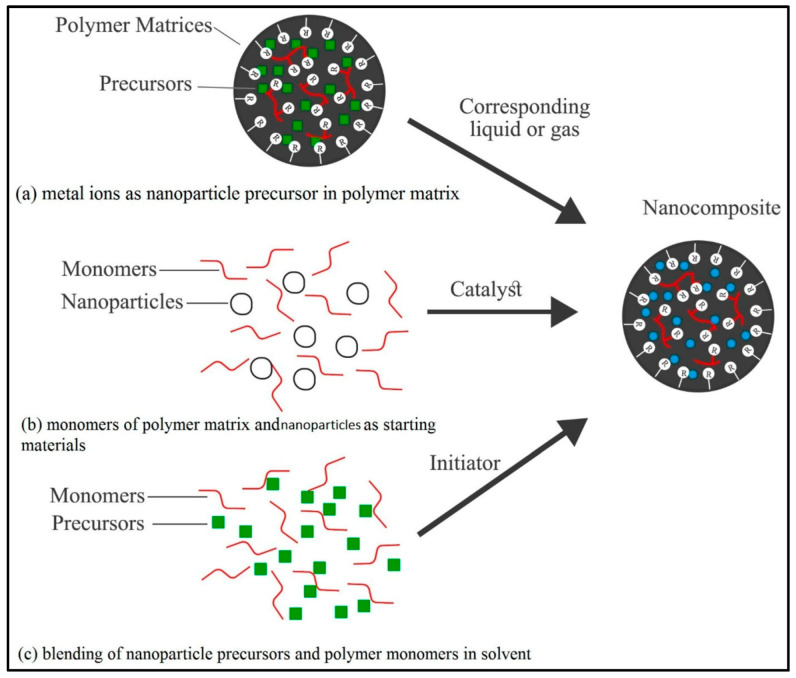
Process for creating mixed-matrix membranes in situ. Reprinted from reference [69].

Some chemicals, like halides and sulfides, may be readily and immediately produced within the polymer matrix. The in situ synthesis method is also employed in the construction of these membranes. This in situ procedure has three methods. These are illustrated in Figure 4 in detail [69].

The following are the main procedures for creating this membrane:

1. Selection of appropriate inorganic nano-additives for uniform dispersion into the membrane matrix;
2. Comprehension of the interaction between polymer and additive for the design of the optimum membrane structure;
3. Improved regeneration or cleaning of mixed-matrix membranes for reuse, as well as an increase in the stability of nano-additives in the membrane structure after production for a long service life [70].

##### Layer-by-Layer Assembly

Layer-by-layer (LbL) can be used to produce membranes with a range of pore sizes and pore distributions. The layer-by-layer assembly method has emerged as a promising manufacturing process for the production of polymeric nano-based membranes. In this approach, the polymer layers are deposited onto a substrate through alternate adsorption of cationic and anionic polymers, resulting in the formation of a dense, uniform, and well-defined membrane.

The LbL assembly method offers several advantages over traditional membrane fabrication methods such as dip-coating, spin-coating, and casting. The LbL assembly process allows for the creation of multilayer membranes of well-defined thickness and composition that can be precisely controlled by selecting polymers and the number of layers deposited. This results in improved performance and functionality compared to single-layer membranes [71].

One of the key benefits of using nanoparticle-embedded polymers fabricated using the LbL assembly method is their enhanced mechanical stability. By incorporating nanoparticles into polymers, the strength and durability of the membranes can be greatly improved for their use in advanced energy and environmental applications. This makes them suitable for use in a range of harsh environments, such as in high-pressure and high-temperature applications [72]. 

Recent advancements in the LbL assembly method have led to the development of new nanoparticle-embedded polymers with enhanced properties. For example, the use of nanoparticles and other nanoscale materials as building blocks has enabled the creation of membranes with improved mechanical strength, permeability, and selectivity. Furthermore, the incorporation of functional groups into the polymers has enabled the design of membranes with specific properties, such as pH sensitivity, biocompatibility, and catalytic activity [73,74,75]. The LbL assembly method also enables the creation of nanoparticle-embedded polymers with controlled drug delivery capabilities. By incorporating functional groups into the polymers, membranes can be designed to respond to specific stimuli, such as changes in pH or temperature, and to release drugs in a controlled manner. This information was reviewed by Park in a study on the layer-by-layer assembly of nanoparticle-embedded polymers for use in controlled drug delivery [76]. The utilization of renewable and biodegradable materials to produce nanoparticle-embedded polymers is also a growing area of research, as highlighted in many studies on the layer-by-layer assembly of green and sustainable polymeric nano-based membranes. This not only offers environmental benefits but also cost-saving potential. This is because biodegradable materials can be produced and sourced more efficiently than traditional petroleum-based polymers [77].

##### Template-Assisted Methods

Template-assisted methods involve the use of a template to produce a polymer membrane with a specific pore size and structure. The template can be a porous material such as a sieve, or a solid material. The polymer is then deposited onto the template; after the removal of the template, a porous membrane is produced.

One popular template-assisted method is the track-etched (or shadow-masked) method. This involves the use of a metal template with microscopic pores that define the shape and size of the membrane [78] and the use of a track-etched method to produce nanoparticle-embedded polymers with uniform and controlled pore sizes. Another template-assisted method is the nano-imprinting method, which involves pressing a polymer film onto a template and then removing the template to obtain the desired pattern. A third template-assisted method is a soft-lithography method, which uses a mold made from a soft, flexible material to create complex patterns and shapes. A study by Wang et al. (2021) explored the use of soft lithography for the production of nanoparticle-embedded polymers with enhanced mechanical stability and permeability [79].

In conclusion, template-assisted methods, including layer-by-layer assembly, electrospinning, chemical vapor deposition, solvent casting, and phase inversion, continue to offer promising avenues for the production of nanoparticle-embedded polymers with improved properties. Ongoing research efforts are focused on exploring new materials and techniques for the production of membranes with a superior performance in various applications.

##### Track-Etching Methods

Track-etching methods for membrane characterization have gained popularity in recent years due to their ability to create uniform and controlled pores in membranes for a variety of applications. These methods include two primary techniques: particle-induced track etching and nuclear track etching. Both techniques use ion beams to create tracks in polymer membranes. Particle-induced track etching involves the use of heavy ions, such as alpha particles or heavy ions, to create tracks in the membrane [80]. On the other hand, nuclear track etching involves the use of fast neutrons to create tracks in the membrane. Once the tracks are created, the membrane is chemically etched to remove the polymer around the tracks, leaving behind uniform and controlled pores in the membrane. The pore size and density can be controlled by adjusting the ion beam energy, the etching time, and the chemical etching conditions [81]. The primary advantage of this method is the ability to create uniform and controlled pores in the membrane [82]. This allows for precise control of pore size, shape, and density. This is critical for many applications, such as filtration, separation, and drug delivery. Additionally, track-etching methods are compatible with a wide range of polymer materials, making them versatile for different applications. 

Although track-etching methods have several advantages, they also have some limitations. One limitation is the inability to create pores smaller than the ion track diameter. This limits the minimum pore size that can be achieved using these methods. Additionally, the ion beam used in these techniques can cause damage to the polymer membrane, leading to degradation or changes in the mechanical properties of the membrane. The chemical etching process can also cause damage to the membrane, leading to irregularities in pore size and shape [83].

Track-etched membranes (TeMs), manufactured from polycarbonate and poly(ethylene terephthalate) (PET), have a wide range of physical, chemical and mechanical properties and are perfect templates for developing composite materials. Varying the irradiation conditions makes it possible to obtain TeMs with pore density, while the modification of the conditions of the etching stage provides the production of membranes with different geometries (cylindrical, cone-shaped, bottle-shaped, etc.) and with pore diameters ranging in size from 50 nm to 5 μm [83]. As we mentioned earlier, the effectiveness of membrane-based separations is limited by the fouling of the membrane used. In order to mitigate this effect on PET track-etched membranes (PET TeMs), the methods of oxidation in hydrogen peroxide- and radiation-induced graft polymerization of acrylic acid (AA) and N-vinylimidazole (VIM) were used. The antifouling properties of the modified PET TeMs showed that oxidation led to improved antifouling properties, whereas graft polymerization of AA and VIM, on the contrary, led to increased fouling of the membrane surface [84].

#### 3.1.2. Homogeneous Nanoparticle-Embedded Polymers (HPMs)

Based on nanomaterials, homogenous polymeric membranes possess better selectivity and higher permeability and are mostly fabricated using surface coating and self-assembly methods. Such applications have been made in the separation of organic molecules and inorganic salts in gas and liquid mixture systems by capitalizing on the better permeability, anti-fouling, stability, and accuracy of the nano-based polymeric membranes [84]. The advantages of HPMs include their high permeability, selectivity, and mechanical stability. Additionally, they are easy to process and can be produced in large quantities. HPMs also have the potential to be modified with functional groups to enhance their performance in specific applications [85,86,87,88].

Researchers are exploring various approaches with which to synthesize and modify these polymers, including the use of copolymers and block copolymers, and general surface modification. There has also been a growing interest in the use of HPMs for various applications, such as desalination, wastewater treatment, and gas separation. The development of polymeric nanoparticles in the last decade has been used as a steppingstone into the discovery, development, and fabrication of HPMs, which theoretically have been presumed to possess excellent performance attributes [89,90]. HPMs are theoretically expected to display performance advantages over conventional MMMs due to the continuous transfer channels formed between the various blocks of materials making up the polymeric membrane [91]. Furthermore, the surface structure and hierarchical nature of HPMs can be adjusted to suit the requirements of the project by adjusting the physicochemical properties of the nanoparticles making up the membrane. HPMs are also constructed using self-assembly and surface coating techniques, which are also used to manufacture mixed-matrix membranes [92].

Polydopamine (PDA) nanoparticles, hyper-branched polymers, and other nanoparticles are used to manufacture homogenous polymeric membranes through surface coating methods [93]. Surface coating in HPMs generation is used to produce elements such as zwitterionic colloid nanoparticles. These, when subjected to further processing, produce zwitterionic colloid nanoparticles membranes (ZCPMs) [94].

In terms of desalination, HPMs are effective at removing salt from water, and they have been shown to have a high salt rejection rate and high water permeability [95]. In the area of wastewater treatment, HPMs have been used to remove organic pollutants and heavy metals from water, and they have shown promising results in terms of their ability to effectively purify contaminated water [96]. For gas separation, HPMs have been used to separate a variety of gases, including nitrogen, oxygen, carbon dioxide, and hydrogen. The unique properties of these materials, such as their high permeability and selectivity, make their use practical in gas separation applications, and they have the potential to be used as alternatives to traditional gas separation methods. Furthermore, the versatility of HPMs has led to the exploration of their use in various other applications, such as biomedicine. HPMs are biocompatible and have been used to develop drug delivery systems and implantable medical devices. Their ability to control the release of drugs, as well as their biocompatibility, makes them suitable for use in these applications. In the field of energy production, HPMs have been used in the development of fuel cells to serve as proton exchange membranes. The high proton conductivity and stability of HPMs make them appropriate for this application, and they have the potential to be used as a more efficient alternatives to traditional fuel cell membranes [97].

Polymeric nanomaterials can also be fabricated using the filtration assembly method, which, in comparison to the surface coating method, can also produce the desired nano-based membranes [98]. In the filtration assembly method, a vacuum-driven or pressure-driven force is used. The polymeric nanoparticles, which are pre-dispersed before the process starts, are used to make the filtration solutions that are used later in the process, and the solution of dispersed nanoparticles is filtered through regulated pores to create the desired films [99]. 

The manufacturing process of HPMs is complex and multi-faceted, with a range of techniques being used to produce membranes with improved properties. Ongoing research efforts are focused on exploring new materials and techniques for the production of HPMs with superior performances in various applications.

##### Surface Coating

Surface coating is a method of depositing a thin layer of polymeric material onto a substrate surface to form a uniform and homogenous membrane. This technique has several advantages over other membrane manufacturing methods, including the ability to produce membranes with a high degree of control over pore size and distribution, and the ability to produce membranes with uniform and consistent properties [100].

The process of manufacturing HPMs using surface coating techniques typically involves substrate preparation, the deposition of polymeric material, cross-linking, characterization, and modification (if necessary). In the first step, the substrate material is cleaned and treated in order to ensure a high-quality and uniform surface. The polymeric material, such as polysulfone or polyvinylidene fluoride, is then deposited onto the substrate using techniques such as dip-coating, spin-coating, or spray-coating. The deposited polymeric material is cross-linked to form a stable and continuous membrane through chemical cross-linking, thermal cross-linking, or UV cross-linking. The final membrane is characterized to evaluate its performance and properties, such as pore size, surface area, and water flux.

Modifications to the membrane can be made by incorporating functional groups or nanoparticles in order to enhance its performance for specific applications [101]. For example, the incorporation of nanoparticles can increase the mechanical strength of the membrane and improve its resistance to fouling.

The use of surface coating techniques to manufacture HPMs has resulted in the production of high-quality and consistent membranes which possess excellent performance and durability. The ability to control pore size and distribution, as well as the ability to modify the membrane surface, has enabled the development of membranes with specific properties for use in various applications.

Surface coating was used to produce HPMs with high selectivity and flux. The authors used a combination of dip-coating and spin-coating techniques to deposit a thin layer of polyvinylidene fluoride (PVDF) onto a porous support, where the resulting HPMs showed excellent performance in the separation of small organic molecules [102]. 

The process involves the use of hyperbranched polymers, and zwitterionic and PDA nanoparticles. In order to improve the surface coating, recent developments are being integrated into existing techniques. These include combining the propylene imine dendrimers that are developed by 16 thiol groups (DAB-3-(SH)16) to produce polymeric modified membranes [103]. Alternatively, dopamine can be electrosprayed in its aqueous solution onto an ultrafiltration substrate membrane in order to produce a PDA membrane. Additionally, through surface coating, zwitterionic nanoparticles are being improved to promote the manufacturing of homogenous polymeric nano-based membranes. 

The improved zwitterionic nanostructure can be separated by adjusting the monomer ratio of the zwitterionic colloid nanoparticles (ZCPs) and regulating the acid concentration. Additionally, the process involves the polyelectrolyte method that assists in classifying different ionic groups. In their study, Clarizia and Bernardo (2022) argue that the surface coating method utilized protection and de-protection methods to identify weak and strong nanoparticles. The homogenous polymeric PECN-based membrane that formed eased the separation of PV and NF elements. Notably, PAA–PDDA PECNs exhibited a strong perm selectivity membrane in the aqueous ethanol solution. The membrane separation ability has recently been improved by combining organic–inorganic hybrid PECNs. Recent developments in inorganic nanomaterials have integrated silica oxide (SiO_2_) into carbon nanotubes. Such an improvement results in the formation of an inorganic nanomaterial combined with a PECN matrix with high perm selectivity and stable thermal capability [103]. Moreover, the homogenous PECN developed is used for industrial and research purposes.

##### Self-Assembly Process

In self-assembly, the polymeric material is dissolved in a solvent and then cast onto a substrate. The solvent evaporates, leaving behind a thin film of polymeric material. The film can then be manipulated to form the desired structure and pore size, leading to the formation of a functional HPM.

This bottom-up methodology can only be used to fabricate single-sided coatings on planar substrates. This method can be easily applied to a wide range of materials without the need for complicated equipment [70]. Additionally, the filtration assembly process can prepare the homogenous polymeric nano-based membrane. The process involves polymeric nanomaterial that is used to prepare filtration elements. Firstly, the isolated solutions are filtered through porous sieves to develop nano-assembled films [104]. Recently, reversed filtration has been used to improve the deposition of PDA particles onto the internal pores of the polymeric membrane. Alternatively, improving polyethersulfone (PES) ultrafiltration membrane leads to the development of charged membranes, crucial in facilitating the assembly of oppositely charged membranes. Notably, the recent development of the assembly method has influenced the development of ferritin-based membranes and thick UF membranes that regulate the polymeric nanomaterials and self-assembled layers.

Despite the improved membranes, most of them are fabricated, hindering ionic separation. To improve the separation, self-assembled membranes are developed from PECN nanomaterials. Sodium vinyl sulfonate and PECN nanomaterials are combined in a vacuum filtration chamber to improve the structure of the membrane [103]. Hence, the thickness of the membrane and structure alignment of the membrane depend on the preparation condition and the size of the particles. The PECN membrane demonstrates a strong thermal and mechanical capability. Moreover, the process involves the use of an electrostatic layer-by-layer assembly method to fabricate the developed PECN membrane. Depending on the industrial use, the membrane thickness can be changed by regulating the number of nano layers used and several layers-to-layer structures. 

##### Interfacial Polymerization (IP)

Interfacial polymerization refers to the chemical reaction that takes place at the interface between two immiscible polymer systems. The objective of interfacial polymerization of HPMs is to synthesize ultrathin polymer films by inducing polymerization reactions at the interface between a monomer solution and a continuous polymer matrix. This process has become increasingly popular in the development of advanced materials, particularly in the field of nano-based membranes. One of the most important steps in the interfacial polymerization process of HPMs is the selection of the monomer and the initiator. The monomer must be water-soluble and have a low viscosity, and the initiator must have a high degree of reactivity in order to ensure a fast polymerization reaction. A commonly used monomer for the synthesis of HPMs is acrylamide, and the initiator is usually a redox system composed of a reducing agent and an oxidizing agent [105].

This technique is mostly used to prepare the reverse osmosis and nanofiltration membranes that are commercially available in the market. In this process, appropriate polymeric nanomaterials are added into reactive solutions of either organic phase or aqueous monomer solutions and mixed through interfacial polymerization [106]. Interfacial polymerization is performed under the regulation of some specific nanomaterials. These include protein nanosheets, and nanocrystals of cellulose, among other additives, which vary according to the desired output. The protein nanosheets usually used in this process should be appropriately porous to enable better control over the process [107].

Hydrophilic nanomaterials, one of the most used nanomaterials in the interfacial polymerization process, are used to create the desired mixed-matrix membranes by adding to the aqueous phase solutions, and the structure and performance of resultant separation membranes can be adjusted by changing some elements of the interfacial polymerization process. Although these nanoparticles used in the interfacial polymerization process improve the performance of the resultant membranes, the process of acquiring and manufacturing these specific nanoparticles is time-consuming, delicate, requires greater attention, and is generally more costly than the tasks of other performing techniques [108]. There are many examples and applications of them:

Thin-film composite nanofiltration membranes are produced by performing an interfacial polymerization process of piperazine and trimesoylchloride on virgin- and nanoparticle (SiO_2_/TiO_2_)-modified substrates. Substrates of 70:30 polyacrylonitrile and 30:70 polyacrylonitrile, relevant for their use in ultrafiltration, are composed of a polyacrylonitrile polyvinylidenefluoride mix. NF membranes constructed on TiO_2_-modified substrates demonstrate greater flux than other membranes. Salts that are moving from divalent to monovalent salts are rejected at the greatest rate (4.63) by membranes produced on TiO_2_-modified 70:30 substrate blends. Th flux recovery ratio (FRR) and total fouling ratio (TFR) values of nanofiltration membranes fabricated on nanoparticle-modified substrates are much greater than those of NF membranes fabricated on unmodified ultrafiltration substrates [19].

In one study, the utilization of a PS/8PVP substrate in the interfacial polymerization process formed a thin, laminate-like polyamide skin layer of 40 nm thickness and a nanoporous, compressible, sponge-like substrate layer (middle layer). The substrate’s symmetric surface pore shape permitted the creation of an ultrathin polyamide rejection layer in the form of a laminate (of almost uniform thickness) with minimal pore penetration. The barrier layer (polyamide), a thin-film composite nanofiltration (TFCNF) membrane, required sufficient stiffness and functionality, something that was ensured by optimizing relative monomer concentrations, polymerization time, and curing conditions (Curing temperature and time). For the barrier layer of this (NF/PS/8PVP) membrane, the O/N ratio was 2.76, and the study indicated that this layer (polyamide layer) consisted of a well-balanced combination of crosslinked segments as well as many linear subunits containing carboxylic acid groups. These facts rendered the membrane surface hydrophilic with a lower contact angle and imparted a negative zeta potential that increased with an increase in the pH. This membrane with a narrow pore size distribution (median pore radius of 0.26 nm) had an outstanding water flow of 158 L M^−2^H^−1^ at 50 pressure and a strong rejection (>99%) of bivalent salt (Na_2_SO_4_) [20].

Additionally, through the incorporation of negatively charged hydrophilic PSS into the polyamide layer during the (IP) process over a PAN membrane substrate, polypiperazinetrimesamide–PSS semi-IPN-based TFC membranes were fabricated. NF membranes had a greater hydrophilicity and surface potential than virgin polypiperazinetrimesamide NF membranes. On a PAN substrate, semi-IPN membranes revealed a nodular surface architecture with a somewhat thinner polyamide layer. These modified membranes demonstrated a 2–2.5-fold increase in flow, salt rejection (%R of Na_2_SO_4_ and MgSO_4_), and rejection ratios of bivalent and monovalent salts compared to the ratios of the virgin polypiperazinetrimesamide membranes. Due to their hydrophilic nature, these membranes prevented protein molecules from adhering to their surface and exhibited superior antifouling capabilities compared to virgin polypiperazinetrimesamide membrane [109].

## 4. Recent Nanoparticle-Embedded Polymers and their Applications

Nanoparticle-embedded polymers have shown great potential in various advanced research applications, including environmental engineering.

Nanoparticle-embedded polymers have been used in water and wastewater treatment applications, where they show high efficiency in removing contaminants such as heavy metals, organic pollutants, and pathogens. The high selectivity and permeability of these membranes make them suitable for the purification of water and the removal of toxic pollutants from industrial effluents. Different technologies are used for this, e.g., membrane bioreactors (MBRs), which are used for the treatment of wastewater and are composed of polymeric nanomembranes. They are effective in removing a wide range of pollutants from wastewater, including organic compounds, heavy metals, and microbes. A chitosan composite is one kind of polymeric enhancement nanoparticle. The reverse micelles, ionic gelation, and precipitation techniques produce chitosan nanoparticles. The synthesized nanoparticles are then integrated into polyethersulfone membranes. Improved water contact angle, permeability, salt exclusion, and antifouling characteristics characterize the process. Research shows that the water contact angle in PES-enhanced membranes is significantly reduced compared to that of a standard membrane. One study has also recorded an 88% salt rejection value [69]. The method is applied in wastewater treatment to sieve out heavy metal material and dye separation.

Nanofiltration (NF) is a process of separating and removing contaminants from aqueous solutions by passing them through a membrane. Nanofiltration membranes are composed of polymeric nanomembranes and are used for the removal of trace organic compounds, heavy metals, and other impurities from water. Reverse osmosis (RO) is a process that uses a semi-permeable membrane to remove dissolved solids and other dissolved impurities from water. Polymeric nanomembranes are used in RO systems to provide a barrier against the passage of large molecules, such as salts and organics. Advanced oxidation processes (AOPs) use polymeric nanomembranes as a medium for catalyzing oxidation reactions in order to degrade pollutants. This process can be used to remove contaminants from water, soil, and air. Polymeric nanomembranes are used for the adsorption of heavy metals from wastewater. The nanomembranes have high adsorption capacities for heavy metals, making them an effective option for the removal of these pollutants from water.

Graphene oxide is a crystalline carbon-based nanoparticle. The graphene oxide part of the materials under discussion is produced through Hummer’s method. The nanoparticles possess a hexagonal unit cell structure. The particles are then amalgamated by double-casting phase inversion. The nanoparticle application enhances membrane permeability, compactness, and antifouling characteristics [110]. It is applied in water desalination to reduce filtration costs because of the compactness of graphene oxide synthesis. The method minimizes membrane interlayer spacing.

Titanium dioxide nanoparticles can enhance the hydrophilicity of polymer membranes. Nanoparticles are synthesized into polymer composites through dry–wet phase inversion and spinning. The nanoparticles strongly bond to membranes and undergo minimal degradation. The enhanced properties in titanium dioxide membranes are increased contact angle and improved porosity [111]. The membranes are widely used in water purification to separate contaminants, and their increased efficiency, thermal properties, and stability can be attributed to the presence of titanium dioxide nanoparticles within the membrane composition.

Silicon dioxide nanoparticles are integrated into hollow polyethersulfone fiber membranes, processed in a sodium dodecyl sulfate solution, brought to the doping stage and synthesized into the membrane by phase inversion. The addition of the nanomaterial promotes thermal stability, pure water flux, and hydrophilicity in composite membranes [112].

An aluminum oxide nanoparticle is a significant metal oxide for the improvement of anti-adhesiveness. It is also coated with zinc oxide by homogenous precipitation. Saraswathi et al. (2019) state that the oxide imparts stronger membranes and increases water flux due to the charge of the zinc oxide coating. The hydrophobic effect of the modified coating promotes anti-adhesiveness in the membrane [111].

Hematite, in conjunction with aluminum hydroxide, is a significant nanoparticle with applications in membrane development. The nanoparticle is formed by hydrothermal synthesis, a process in which crystal synthesis is conducted under pressure. The resulting solution is determined to have high adsorption and turbidity characteristics [113]. The hematite membrane product is implemented in boilers as a corrosion inhibitor.

Nanoscale zero-valent iron is a nanomaterial instituted to enhance membrane performance. The reduction of dissolved iron particles fabricates the nanoparticle. Electrospinning is performed to prevent agglomeration and improve porosity. The synthesis of the nanomaterial is characterized by uniformity, strong adhesion, antifouling, chemical stability, and permeability [111]. The material is applied in precipitation and the adsorption of pollutants in water.

Nanoparticles of calcium carbonate are utilized to produce ceramic membranes. The process is performed by a precipitation reaction of calcium chloride and sodium carbonate with a high-pressure jet injection. The resulting products feature improved permeability, stability, and increased contact angle. Maleki et al. (2019) state that adding nanoparticles to the membrane past a limit causes undesirable characteristics. Calcium carbonate is utilized as an economical filler in polymer synthesis [114].

When carbon nanotubes are integrated into membranes, their porosity, hydrophobicity, chemical stability, foul resistance, and surface roughness are observably enhanced features [115]. This membrane is utilized in a separation process.

Montmorillonites are polymer-based nanoparticles obtained from soft mineral elements. The product is soft, porous, and bendable. Montmorillonite is applied in engines and industrial water treatment due to its suitable filtration properties [111].

Cellulose nanocrystals are derived from cellulose fiber by hydrolysis. The material is made by grinding and centrifuging to separate the nanocrystals from the fibers. The desirable features offered by these nanoparticles include hydrophilicity, non-toxicity, and enhanced strength [116]. Membranes developed from cellulose nanocrystals are used in water treatment plants, especially for filtration.

Polymeric nano-based membranes are explored for their potential in energy generation and storage applications. For example, they are used in fuel cells to improve the performance and stability of the electrocatalysts, and in supercapacitors as high-performance electrodes for energy storage. 

Zirconium is a stable nanocomposite used in membrane development. It is used in the enhancement of the Nafion membrane. The nanoparticle synthesis is performed by phase inversion, which is initiated by boiling hydrogen peroxide to remove contaminants. It is then subsequently moved to a sulfuric acid and immersed in distilled water. The product is then integrated into a membrane via methanol pre-soaking and fusion. The material is defined by low toxicity, stability, improved liquid uptake, hydroxide ion conductivity, and hydrophilicity [117]. The zirconium-enhanced nanocomposite has applications in alkaline fuel cells because of its stability in hydrogen–oxygen surroundings. Zhang et al. observed improvements in porosity in addition to hydrophilicity and low toxicity. Due to the semiconductor’s desirable attributes, it is applied in micro conductors, solar cells, battery cells, nanotubes, and nanofibers [118].

Polymeric nano-based membranes have been utilized as sensing platforms in various areas, such as environmental monitoring, food safety, and medical diagnostics. They can be functionalized with specific recognition elements, such as antibodies or enzymes, to detect target analytes in complex matrices. Cellulose nanofibrils are rod-like nanocomposites with improved mechanical properties. The nanomaterial is harvested by the hydrolysis of enzymes. The nanoparticle possesses high strength and stiffness, low weight, and improved biocompatibility [116]. Due to their high strength, nanofibrils are utilized in filtering out heavy metals in water treatment. Polysulfone nanomembranes have many properties that make them suitable for use in blood filtration in the process of hemodialysis, in the recycling of wastewater, and in gas separation chambers. One of the recently developed applications is the integration of iron nanoparticles with membranes so that the membranes gain additional properties. The size of magnetic nanoparticles in the membrane is a significant factor affecting the membrane properties and should therefore be considered [113]. 

The polymeric membrane can separate gases successfully. Compared to absorption, cryogenic distillation, or other processes, it has several advantages such as being easier to operate, energy-efficient, and having a smaller environmental effect [119,120]. Additionally, gas separation by the polymeric membrane is one of the most promising technologies for the petrochemical and chemical industries as well as environmental engineering. Several recent studies have focused on enhancing membrane’s efficiency [121,122,123,124,125]. Emerging nanoparticles possess new potential for incorporation into polymeric material for the fabrication of GS/PV membranes with better performance and properties [126]. High-performance GS/PV polymeric membranes can be achieved by incorporating nanomaterials using physically doped and chemically bonded techniques [127,128,129].

Nanomaterials like covalent organic frameworks (COFs) and metal–organic framework (MOFs) have been extensively explored to improve polymeric membrane qualities because of their beneficial traits, such as high stability, high porosity, and tunable surface properties and structure [126,130,131,132]. COFs are another class of excellently performing porous crystalline materials. In addition, the compatibility of COFs with polymers contributes to their high dispersibility in polymer membranes. In addition to that, MOFs are an essential new material for membrane technology, particularly in gas separation and pervaporation, because of their desired pore size and extra channels [126].

Zeolites are used in gas membrane development. They are modified via a casting method in which silver ions are integrated into the zeolite material to form a membrane. The film is covered with a thin layer of polydimethylsiloxane, where the cations in silver promote the self-transportation of gases. Zeolite-enhanced films exhibit good thermal and chemical stability as well as selective gas separation. Improved zeolite membranes are applied where liquids and gases of similar molecule size are separated due to the embedded nanoparticles which improve the separation process [93]. For an example of gas separation, one study saw a mixed-matrix composite membrane (MMCM) containing NH2-ZIF-8 nanocrystals be successfully manufactured by designing polyimide/MOF interfacial hydrogen or covalent bonds, which lowered the selective layer to 280 or 140 nm in thickness. The protruding MOF filler can significantly lower transport resistance and enhance CO_2_ diffusion. Consequently, MMCMs have a high CO_2_ permeance of 778 GPU and a CO_2_/CH_4_ selectivity of 34 [133].

COFs can also work in conjunction with nanomaterials to enhance the gas separation performance of composite membranes. CTF-BTD/GO membranes, for instance, were produced by depositing CTF-BTD and GO nanosheets onto anodic aluminum oxide (AAO) substrate surfaces. The H_2_ permeation and H_2_/CO_2_ selectivity of the CTF-BTD/GO membranes were 655.6 GPU and 43.1, respectively [134].

Since hydrogen is one of the key chemical sources for industries and clean energy, technologies that separate hydrogen have gained increasing relevance in recent years. In addition to being a useful fuel, it is also a significant contributor to the production of electricity and other forms of useful energy [120,135]. To obtain hydrogen of high purity, the separation of hydrogen from carbon dioxide is a crucial step [120,136]. Due to their excellent hydrogen selectivity, palladium membranes have been researched extensively for use in hydrogen separation. However, the use of palladium and its alloys has some drawbacks, including high sensitivity to chemicals (i.e., sulfur, chlorine, and carbon monoxide) and an exceedingly expensive price [120,137,138,139].

The nanomaterial of palladium–iron possesses significant hydrogen–helium selectivity. The modification technique applied in the material is ion-exchange pore diffusion between iron and palladium. The product is highly resistant to cracking, is thermally stable, and possesses hydrogen flux [111]. 

Establishing a wastewater reuse technology is necessary as the population rises and more people depend on fewer water sources due to sources being polluted or unfit for human use. Nanotechnology offers several advantages for the development of advanced membranes for water treatment. First, nanoporous membranes can offer high selectivity, which can lead to high rejection rates for specific solutes. Second, the incorporation of nanomaterials can improve membrane properties such as permeability, durability, and fouling resistance. Third, nanotechnology can provide a platform for the development of smart membranes with the capacity to respond to changes in the feed water quality, such as pH, temperature, and ionic strength. Filtration technologies play a very important role in the use of membrane technology. Membrane filtration is a method of physical and chemical purification that can be used to remove contaminants [140]. Therefore, it is important that researchers create antibacterial nanocomposite membranes with additional hydrophilic qualities for use in water reuse and wastewater treatment. Polyvinyl chloride (PVC) flat-sheet membranes are manufactured using the phase inversion method which introduces nanoparticles of silver oxide (Ag_2_O) into the membranes. In one study, as an important feature for the use of this type of membrane in a holding company of water and wastewater purification, the mechanical strength of the modified PVC membrane created with Ag_2_O was high, with a Young’s modulus of 5.5 MPa, compared to the modulus of the pristine PVC membrane, which was 3.5 MPa [140]. Future research in the field of using membranes in water treatment should focus on addressing these limitations and developing scalable and cost-effective methods for the fabrication of advanced nanomembranes.

## 5. Future Applications

**Figure 5:**
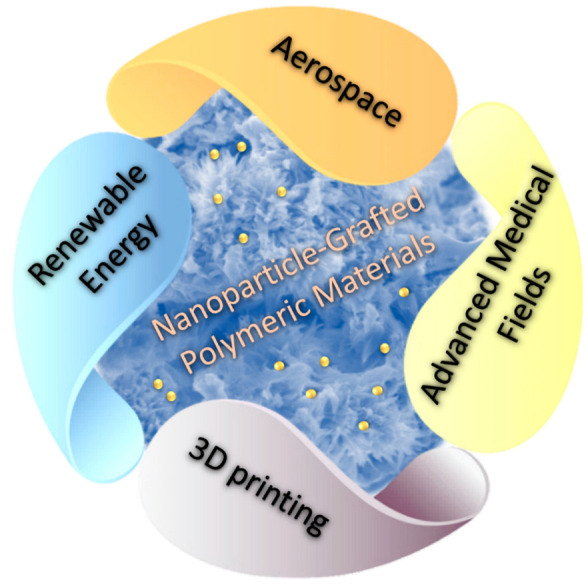
The future application of Nanoparticle-Grafted Polymeric Materials.

Many areas can be considered for the advanced uses of nanoparticle-grafted polymeric materials, as shown in Figure 5, due to the advantages that these materials can possess, with the possibility of obtaining new properties. As we mentioned earlier, the process of nanoparticle embedding can change and improve the properties of the membranes. In the future, we may be able to fabricate grafted polymers with unique levels and properties, especially when more than one component is incorporated when forming the membrane, or when noble materials or elements are used. In this regard, we expect grafted membranes to have advanced applications in aerospace, as well as in artificial intelligence, 3D printing, renewable energy, medical fields, and many more advanced areas. 

The electronics industry is one of the primary areas in which nanoparticle-embedded polymers are expected to have widespread use. The unique combination of suitable bonds provided by the polymers embedded in the nanoparticles makes them suitable for developing high-performance electronics. For example, silver nanoparticles embedded in a polymer matrix can be used to create flexible and stretchable conductive materials [141,142]. This property is desirable in applications such as wearable electronic devices, where the material needs to be flexible and conform to the shape of the human body.

## 6. Conclusions

Nanoparticle-embedded polymers hold significant promise in various fields of application, including electronics, biomedicine, energy, and environmental science. The unique combination of properties offered by nanoparticle-embedded polymers makes them suitable for developing high-performance materials and devices. As research in this field continues to advance, it is expected that the applications of nanoparticle-embedded polymers will continue to expand, leading to the development of new and innovative technologies. In the recent past, there has been rapid growth in the study and development of polymeric nano-based membranes, and more technologies and techniques in the separating industry continue to be innovated and developed. Two kinds of such membranes have been developed for use today due to their varying properties and uses. These are the mixed-matrix membranes, commonly known as MMMs, and the homogenous polymeric nano-based membranes.

Manufacturing processes for nanoparticle-embedded polymers are diverse and constantly evolving, and the choice of method depends on the specific application and desired properties of the material. Physical methods such as electrospinning involve the physical manipulation of the nanoparticles and polymer matrix to create the desired structure. Chemical methods involve the chemical modification, such as in situ polymerization, emulsion polymerization, and covalent attachment, of the nanoparticles or polymer matrix to facilitate the formation of the desired structure.

To improve their performances in selectivity and permeability, various techniques have been developed to alter both the internal structures and the surface characteristics of nano-based membranes. The improvement of performance has seen a rise in the use of nano-based membranes in various fields such as water treatment, biomedical research, the purification of chemical products, the purification of petroleum, and the separation and purification of gaseous substances.

The use of synthetic polymeric nanoparticles to fabricate separation membranes has proven to be expensive and requires cutting-edge technology if it to develop further. Additionally, although the resultant membranes show improved performance in selectivity and permeability, their effectiveness is still below that of membranes developed using molecular channels. The development of these nanoparticles for the enhancement of membranes should also focus on developing the stability of the membranes against extreme temperatures, alkalis, pressure, and other substances. The nanoparticles should also be developed to improve the control of users over both the surface and internal characteristics of the membranes, such as the pores sizes and the length of the channels.

With the level of interest and research that have gone into polymeric membrane development, it is expected that specialized membranes will be developed using nanotechnology soon, and that the scope of use of such membranes will be broadened.

## Data Availability

All data are available.
